# Interference on Cytosolic DNA Activation Attenuates Sepsis Severity: Experiments on Cyclic GMP–AMP Synthase (cGAS) Deficient Mice

**DOI:** 10.3390/ijms222111450

**Published:** 2021-10-23

**Authors:** Peerapat Visitchanakun, Warerat Kaewduangduen, Awirut Chareonsappakit, Paweena Susantitaphong, Prapaporn Pisitkun, Patcharee Ritprajak, Natavudh Townamchai, Asada Leelahavanichkul

**Affiliations:** 1 Department of Microbiology, Faculty of Medicine, Chulalongkorn University, Bangkok 10330, Thailand; peerapat.visitchanakun@gmail.com (P.V.); porpluemw@gmail.com (W.K.); Awirut.turk@gmail.com (A.C.); 2 Translational Research in Inflammation and Immunology Research Unit (TRIRU), Department of Microbiology, Chulalongkorn University, Bangkok 10330, Thailand; 3 Nephrology Unit, Department of Medicine, Faculty of Medicine, Chulalongkorn University, Bangkok 10330, Thailand; pesancerinus@hotmail.com; 4 Research Unit for Metabolic Bone Disease in CKD Patients, Faculty of Medicine, Chulalongkorn University, Bangkok 10330, Thailand; 5 Division of Allergy, Immunology, and Rheumatology, Department of Medicine, Faculty of Medicine, Ramathibodi Hospital, Mahidol University, Bangkok 10330, Thailand; beepisitkun@gmail.com; 6 Research Unit in Integrative Immuno-Microbial Biochemistry and Bioresponsive Nanomaterials, Department of Microbiology, Faculty of Dentistry, Chulalongkorn University, Bangkok 10330, Thailand; p.ritprajak@gmail.com; 7 Renal Immunology and Renal Transplant Research Unit, Department of Medicine, Faculty of Medicine, Chulalongkorn University, Bangkok 10330, Thailand

**Keywords:** sepsis, cell free DNA, LPS, cGAS, cecal ligation and puncture

## Abstract

Although the enhanced responses against serum cell-free DNA (cfDNA) in cases of sepsis—a life-threatening organ dysfunction due to systemic infection—are understood, the influence of the cytosolic DNA receptor cGAS (cyclic guanosine monophosphate–adenosine monophosphate (GMP–AMP) synthase) on sepsis is still unclear. Here, experiments on cGAS deficient (cGAS^-/-^) mice were conducted using cecal ligation and puncture (CLP) and lipopolysaccharide (LPS) injection sepsis models and macrophages. Severity of CLP in cGAS^-/-^ mice was less severe than in wildtype (WT) mice, as indicated by mortality, serum LPS, cfDNA, leukopenia, cytokines (TNF-α, IL-6 and IL-10), organ histology (lung, liver and kidney) and spleen apoptosis. With the LPS injection model, serum cytokines in cGAS^-/-^ mice were lower than in WT mice, despite the similar serum cfDNA level. Likewise, in LPS-activated WT macrophages, the expression of several mitochondria-associated genes (as revealed by RNA sequencing analysis) and a profound reduction in mitochondrial parameters, including maximal respiration (determined by extracellular flux analysis), DNA (mtDNA) and mitochondrial abundance (revealed by fluorescent staining), were demonstrated. These data implied the impact of cfDNA resulting from LPS-induced cell injury. In parallel, an additive effect of bacterial DNA on LPS, seen in comparison with LPS alone, was demonstrated in WT macrophages, but not in cGAS^-/-^ cells, as indicated by supernatant cytokines (TNF-α and IL-6), M1 proinflammatory polarization (iNOS and IL-1β), cGAS, IFN-γ and supernatant cyclic GMP–AMP (cGAMP). In conclusion, cGAS activation by cfDNA from hosts (especially mtDNA) and bacteria was found to induce an additive proinflammatory effect on LPS-activated macrophages which was perhaps responsible for the more pronounced sepsis hyperinflammation observed in WT mice compared with the cGAS^-/-^ group.

## 1. Introduction

A life-threatening organ dysfunction that occurs as a response to systemic infection (sepsis) is a global healthcare problem [1]. The immune responses against both pathogen-associated molecular patterns (PAMPs) and damage-associated molecular patterns (DAMPs) made by organisms and host cells, respectively, during sepsis result in excessive, uncontrolled inflammation [2], which is one of the main causes of sepsis death [3]. The pathophysiology of sepsis hyperinflammation is largely explained by the vigorous reactions of innate immune cells, including macrophages [4]. Indeed, macrophages are the sentinel immune cells found in nearly all organs that are important for the direction of immune responses in sepsis [5]. Among several sepsis immune activators, serum cell-free DNA (cfDNA) is derived from host cells, including mitochondrial DNA (mtDNA) and nuclear DNA (nDNA), and organisms (microbial DNA), and is associated with the enhanced mortality rate of sepsis due to sepsis-induced profound cell-death in either host cells or organisms [6,7,8]. While phagocytosis of cfDNA activates endosomal TLR-9 [9], cfDNA could induce TLR-4 on cell membranes [10] and stimulate cytosolic cyclic GMP–AMP synthase (cGAS) in cell cytoplasm [11,12,13]. Indeed, the activation of cGAS—a recognition receptor for double-stranded DNA (dsDNA)—generates cyclic GMP–AMP (cGAMP)—a secondary messenger of STING (the stimulator of interferon genes)—which promotes inflammatory responses through interferon regulatory factor 3 (IRF3) and nuclear factor κB (NF-κB) signaling [14]. Although cGAS is naturally prepared for the recognition of intracellular organisms (bacteria and viruses), it can also recognize host DNA and DNA from extracellular bacteria [15,16].

Regarding bacterial control, cGAS has a limited role in sepsis because of the cGAS-dependent anti-bactericidal effect against only intracellular bacteria [17,18,19]. It is also not necessary for an anti-microbial effect toward other more common bacteria [18,20], possibly due to other cytosolic DNA receptors [21]. Among cytosolic DNA receptors, the stimulator of interferon genes (STING)-dependent pathway is the main signaling pathway, and the cGAS–STING axis is a major response, especially in sepsis [22,23,24], partly because of the ability to recognize both microbial DNA and self-DNA (a higher concentration activating the threshold for self-DNA) [17]. For bacterial DNA in sepsis, the breakdown of viable organisms in the blood and the translocation of intestinal pathogens from the gut into the blood [25,26] initiates bacterial cfDNA—unmethylated cytosine–phosphate–guanine (CpG) DNA motifs—which through systemic circulation potently activate several immune cells (63–65), leading to the proposal that it be used as a sepsis biomarker [27]. Despite a mild inflammatory induction of CpG DNA injection in mice, CpG DNA synergistically worsened the LPS injection model through several NF-κB mediated cytokines [28,29,30]. Moreover, bacterial free DNAs remain in the serum of patients, despite the administration of antibiotics [31,32], and are also found in patients with non-bacterial sepsis [33]. In polymicrobial sepsis with antibiotic treatment, bacterial breakdown initiates the exposure of several PAMPs, including endotoxin and bacterial cfDNA, which may synergistically activate immune responses [32,34]. As such, the beneficial effect of blood purification in sepsis [35] is, in part, possibly due to reduced levels of DNAemia (presence of cfDNA in the blood) [36].

With regard to self-DNA, sepsis also induces mitochondrial damage, and host cell death enhances free mtDNAs and nDNAs that could be exposed to cytosolic cGAS, thereby facilitating sepsis hyperinflammation in a vicious cycle [37]. Subsequently, severe sepsis inflammation, partly due to cGAS activation, causes further cell death, which more prominently elevates free mtDNA [38,39]. Then, cfDNA, from either bacteria or host cells, might affect sepsis severity, so that the manipulation of cfDNA in sepsis could have beneficial effects. Due to sepsis increasing serum cfDNAs and mtDNAs [40], which promote inflammation through cGAS activation [14,15,41], and with injection of bacterial DNA with LPS worsening sepsis severity [28], the fact that blockage of cGAS downstream-signaling, STING and Interferon regulatory factor 3 (IRF3) attenuates sepsis [23] suggests that the exploration of sepsis in cGAS^-/-^ mice and macrophages would be worthwhile, especially considering the limitedness of research into sepsis in cGAS deficient (cGAS^-/-^) mice. Hence, we produced sepsis models, using cecal ligation and puncture (CLP) and LPS injection, and examined in vitro cGAS^-/-^ macrophages.

## 2. Results

### 2.1. Less Severe Sepsis Was Observed in the Cecal Ligation and Puncture Model and the LPS Injection Model with cGAS Deficiency (cGAS^-/-^) Compared with Wildtype Mice

**Figure 1:**
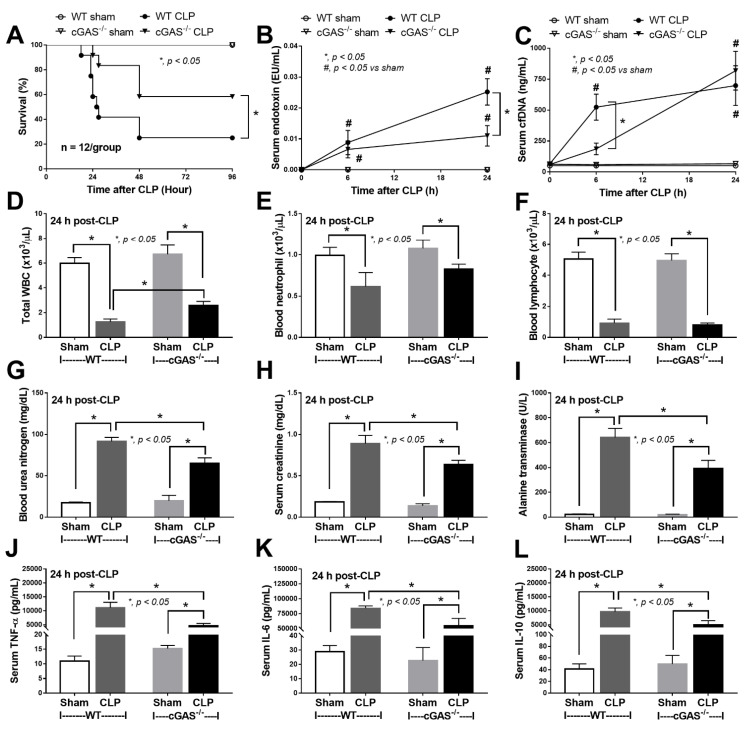
Characteristics of wildtype (WT) and cyclic GMP–AMP synthase deficient (cGAS^-/-^) mice after sham or cecal ligation and puncture (CLP), as indicated by survival analysis (**A**), serum endotoxin (**B**), cell free DNA (cfDNA) (**C**), peripheral blood leukocyte (total, neutrophils and lymphocytes) (**D**–**F**), renal function (blood urea nitrogen and serum creatinine) (**G**,**H**), alanine transaminase (**I**) and serum cytokines (TNF-α, IL-6 and IL-10) (**J**–**L**) (*n* = 12/group for (**A**) and *n* = 7–9/time-point or group for (**B**–**L**)).

**Figure 2:**
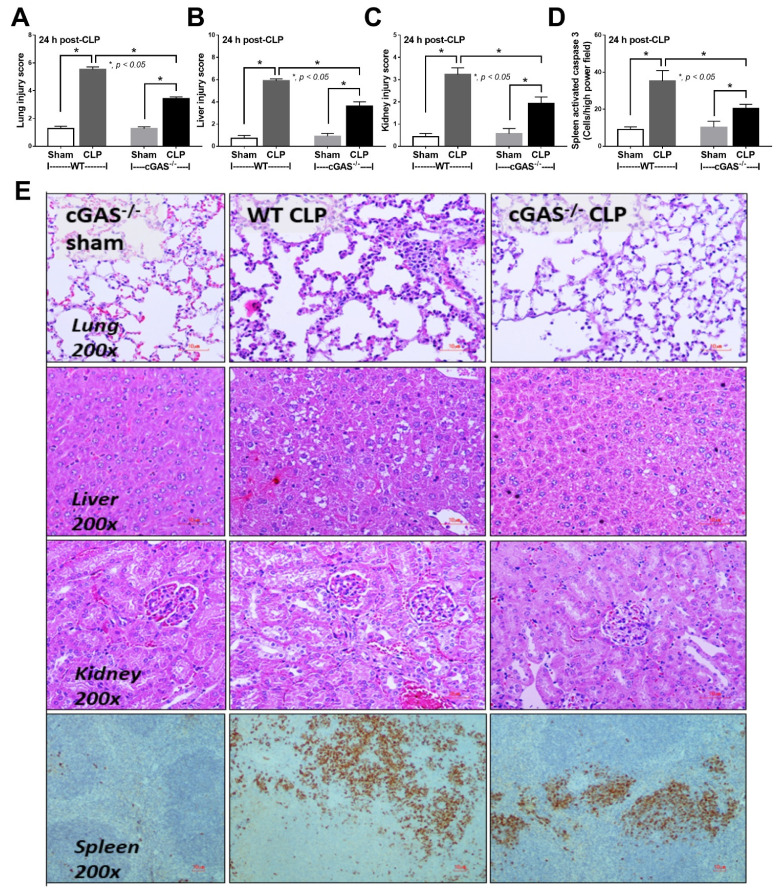
Characteristics of wildtype (WT) and cyclic GMP–AMP synthase deficient (cGAS^-/-^) mice after sham or cecal ligation and puncture (CLP), as indicated by pathological score of lung liver and kidney (**A**–**C**), with activated caspase 3 apoptotic cells in the spleen (**D**) and representative histopathology on H&E staining (lung, liver and kidney) and activated caspase 3 immuno-histochemistry (spleen) (**E**) (*n* = 6–8/group).

**Figure 3:**
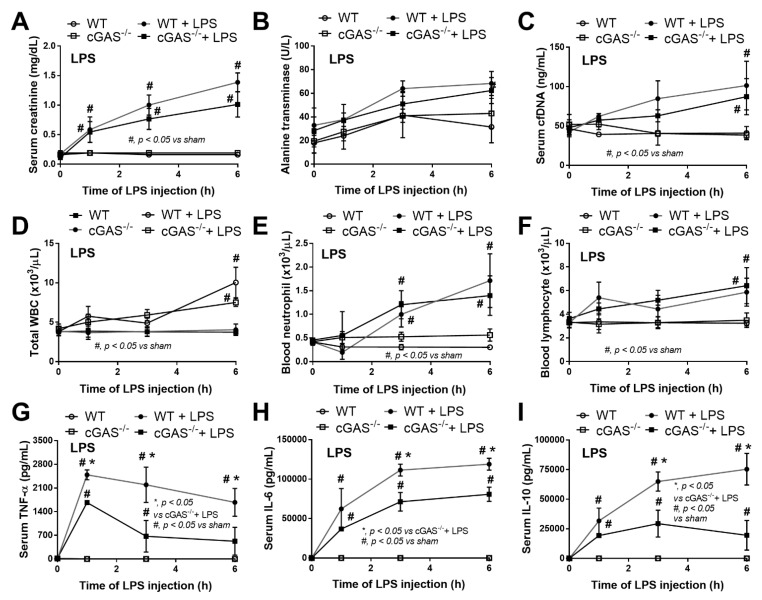
Characteristics of wildtype (WT) and cyclic GMP–AMP synthase deficient (cGAS^-/-^) mice after an injection by normal saline or lipopolysaccharide (LPS), as indicated by serum creatinine (**A**), alanine transaminase (**B**), cell free DNA (cfDNA) (**C**), peripheral blood leukocyte (total, neutrophils and lymphocytes) (**D**–**F**) and serum cytokines (TNF-α, IL-6 and IL-10) (**G**–**I**) (*n* = 6–8/time-point).

Mouse models of sepsis, including cecal ligation and puncture (CLP) and LPS injection, were used to test an impact of cGAS on sepsis severity. Accordingly, cGAS^-/-^ mice exhibited less severe sepsis than the wildtype (WT) mice, as indicated by survival analysis, endotoxemia at 24 h (but not at 6 h post CLP) and serum cell-free DNA (cfDNA) at 6 h (but not at 24 h post CLP) (Figure 1A–C). Additionally, CLP induced leukopenia, neutropenia and lymphopenia in both WT and cGAS^-/-^ mice (Figure 1D–F), but leukopenia was more prominent in WT than cGAS^-/-^ mice, consistent with the more severe sepsis-induced leukopenia in WT mice [42]. Furthermore, renal injury (blood urea nitrogen and serum creatinine), liver damage (serum alanine transaminase), inflammatory cytokines (TNF-α, IL-6 and IL-10), H&E staining histological score (lung, liver and kidney) and spleen apoptosis in WT mice with CLP were more pronounced than in cGAS^-/-^ mice (Figure 1G–L and Figure 2A–E). In the LPS injection model, cGAS^-/-^ mice demonstrated less severe inflammatory responses, as indicated by serum cytokines (TNF-α, IL-6 and IL-10) but not serum creatinine, alanine transaminase, cfDNA and peripheral blood leukocytes (Figure 3A–I). Notably, there was no mortality in LPS-administered mice with the current dose of injection (data not shown).

### 2.2. Less Prominent Inflammatory Responses against LPS in cGAS Deficient (cGAS^-/-^) Macrophages Compared with Wildtype Cells

**Figure 4:**
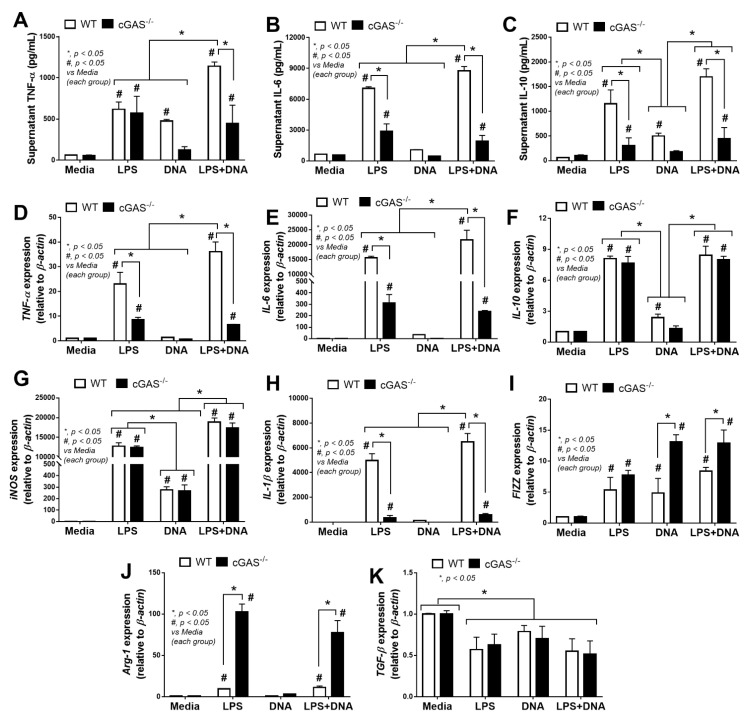
Characteristics of macrophages from wildtype (WT) and cyclic GMP–AMP synthase deficient (cGAS^-/-^) mice after an activation by media control, lipopolysaccharide (LPS), bacterial DNA (DNA) and LPS with DNA (LPS + DNA), as indicated by supernatant cytokines (TNF-α, IL-6 and IL-10) (**A**–**C**) and the expression of genes for cytokines (*TNF-α, IL-6* and *IL-10*) (**D**–**F**), M1 macrophage polarization (*iNOS* and *IL-1β*) (**G**,**H**) and M2 macrophage polarization (*FIZZ-1, Arg-1* and *TGF-β*) (**I**–**K**). Independent triplicated experiments were performed.

Because immune responses against LPS is enhanced by cfDNA, especially bacterial DNA, in the blood [43], inflammatory responses and cell energy status between WT and cGAS^-/-^ macrophages might be different. In WT macrophages, lipofectamine-delivered bacterial DNA (DNA) with LPS stimulation (LPS + DNA) induced more prominent inflammation than LPS alone, as indicated by TNF-α and IL-6 (supernatant cytokines and gene expression) and pro-inflammatory M1 macrophage polarization (expression of *iNOS* and *IL-1β*), while IL-10 (cytokine and gene expression) and anti-inflammatory M2 macrophage polarization (expression of *FIZZ-1*, *Arg-1* and *TGF-β*) were not different between these groups (Figure 4A–K). These data support an additive proinflammatory impact of bacterial DNA in LPS-stimulated macrophages [28,29,30].

**Figure 5:**
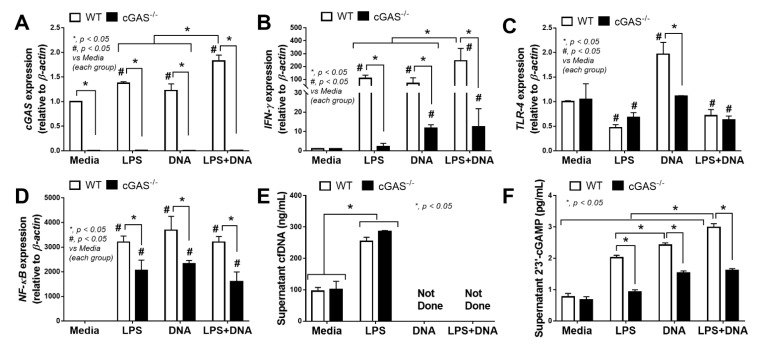
Characteristics of macrophages from wildtype (WT) and cyclic GMP–AMP synthase deficient (cGAS^-/-^) mice after an activation by media control, lipopolysaccharide (LPS), bacterial DNA (DNA) and LPS with DNA (LPS+DNA), as indicated by the expression of *cGAS* (**A**), *IFN-γ* (**B**), *TLR-4* (**C**), *NF-κB* (**D**), supernatant cell free DNA (cfDNA) (**E**) and supernatant cyclic guanosine monophosphate–adenosine monophosphate (cGAMP) (**F**). Independent triplicated experiments were performed. Notably, cfDNA analysis in the DNA alone and LPS + DNA groups was not performed due to the possible interference of bacterial DNA in cfDNA measurement.

In parallel, mild inflammatory activation by bacterial DNA transfection without LPS in WT macrophages was demonstrated by an increase in TNF-α (cytokine), IL-10 (cytokine and gene expression) and *iNOS* expression lower than LPS activation alone (Figure 4A,C,F,G). Meanwhile, compared with LPS alone, an additive inflammatory effect of LPS + DNA was observed in cGAS^-/-^ macrophages with respect to *iNOS* but not with respect to other parameters, despite a synergy of LPS + DNA over LPS alone with respect to several parameters in WT macrophages (Figure 4A–K). Moreover, all these pro-inflammatory parameters, except for *iNOS* and *IL-10*, in cGAS^-/-^ macrophages were lower compared with WT macrophages given all stimulations (Figure 4A–H). In contrast, the M2 macrophage polarization markers (*FIZZ-1* and *Arg-1*, but not *TGF-β*) in cGAS^-/-^ macrophages subject to most of the stimulating conditions were higher compared with WT cells (Figure 4I,J). For the downstream activation, all stimulations (LPS, DNA and LPS + DNA) on WT macrophages upregulated *cGAS* and *IFN-γ*, with higher levels observed in the LPS + DNA group (Figure 5A,B). In parallel, DNA stimulation alone enhanced *TLR-4*, while both LPS and LPS + DNA down-regulated *TLR-4* (Figure 5C), possibly by being associated with TLR-4 internalization (endocytosis) after LPS activation [44]. On the other hand, *NF-κB* expression in WT macrophages after subjection to all stimulations were similar (Figure 5D), despite an additive effect on *cGAS* and *IFN-γ* expression after LPS + DNA compared with LPS alone (Figure 5A,B). These data imply an impact of other transcriptional factors downstream from cGAS [45]. In cGAS^-/-^ macrophages, the expression of all interested genes (except for *TLR-4* after LPS and LPS + DNA) were lower compared WT macrophages (Figure 5A–D). Bacterial DNA transfection upregulated *IFN-γ* in cGAS^-/-^ macrophages (Figure 5B), implying an influence of non-cGAS cytosolic DNA receptors. An addition of LPS on DNA stimulation (LPS + DNA) did not further enhance *IFN-γ* expression in cGAS^-/-^ macrophages in a way that was different from its effect on WT cells (Figure 5B), suggesting an impact of cGAS on LPS-bacterial DNA synergy. Additionally, LPS increased supernatant cfDNA from both strains of macrophages (Figure 5E), supporting an impact of LPS on the initiation of cfDNA expression and the possibility of an immune activation by cfDNA after LPS activation (without bacterial DNA transfection) [10]. Notably, the more profound effect of WT macrophages, when compared with cGAS^-/-^ cells, might be due to the higher production of cGAMP, a product from cGAS activation [11,12,13], in WT cells subject to all activating conditions (Figure 5F); however, the most pronounced responses were observed in LPS + DNA WT macrophages (Figure 5F).

### 2.3. Less Prominent LPS-Induced Mitochondrial Injury in cGAS Deficient (cGAS^-/-^) Macrophages Compared with Wildtype Cells

**Figure 6:**
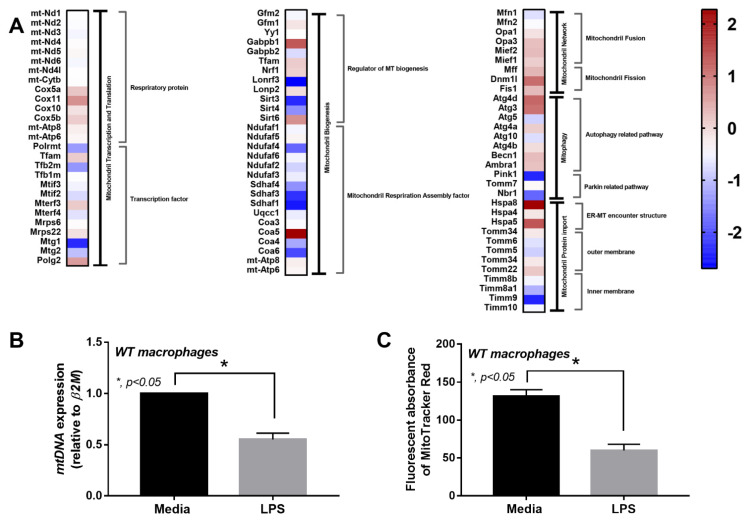
Characteristics of macrophages from wildtype (WT) mice after an activation by media control or lipopolysaccharide (LPS), as indicated by the heat-map of mitochondria-associated genes from RNA sequencing analysis (**A**) and mitochondrial abundance using mitochondrial DNA (mtDNA) and MitoTracker fluorescent staining (**B**,**C**). Independent triplicated experiments were performed. MT, mitochondria; ER-MT, endoplasmic reticulum-mitochondria.

**Figure 7:**
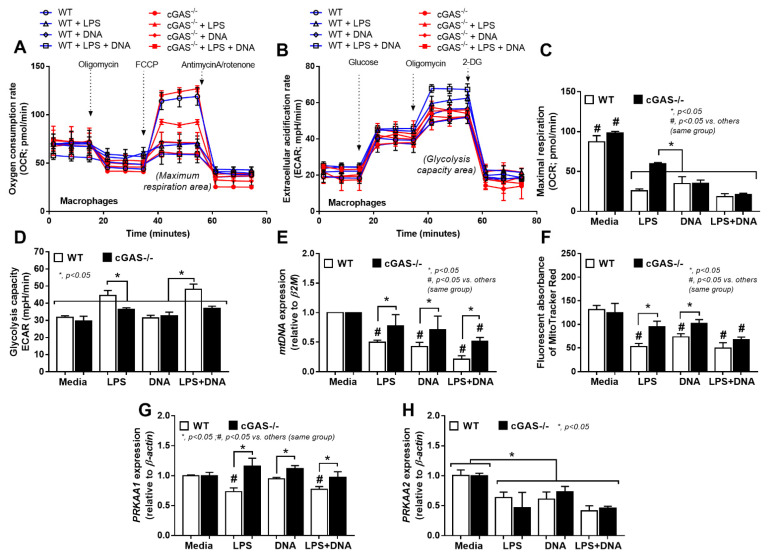
Characteristics of macrophages from wildtype (WT) and cyclic GMP–AMP synthase deficient (cGAS^-/-^) mice after an activation by media control, lipopolysaccharide (LPS), bacterial DNA (DNA) and LPS with DNA (LPS + DNA), as indicated by oxygen consumption rate (OCR) (as determined by mitochondrial stress test) and extracellular acidification rate (ECAR) (determined by glycolysis stress test) (**A**,**B**), along with the graph presentation of maximal respiration and maximal glycolysis (**C**,**D**), mitochondrial abundance (using mitochondrial DNA (mtDNA) and MitoTracker fluorescent staining) (**E**,**F**) and expression of *PRKAA1* and *PRKAA2* (mitochondrial promotors) (**G**,**H**). Independent triplicated experiments were performed.

Due to (i) the role of mitochondria in cell energy status [46] and macrophage responses [47] and (ii) LPS associated cell-energy alteration [48], LPS might have a direct impact on mitochondria. Indeed, there were several upregulated mitochondria-associated genes in several pathways of LPS-activated WT macrophages detected by RNA sequencing analysis, including pathways involved in mitochondrial biogenesis, fission/fusion, autophagy and the production of endoplasmic reticulum encounter structures (Figure 6A). Likewise, the reduction of mitochondria after LPS activation was also demonstrated by the decrease in mtDNA expression and fluorescent-stained mitochondria (Figure 6B,C). In WT macrophages, both LPS and LPS + DNA similarly decreased mitochondrial activity (maximal mitochondrial capacity, determined by extracellular flux analysis), mitochondrial abundance (mtDNA expression, revealed by fluorescent staining) and mitochondrial promoter 5’-AMP-activated protein kinase catalytic subunit alpha (*PRKAA*) subunit 1 and 2 [49], while glycolysis activity (glycolytic capacity), by contrast, increased (Figure 7A–H). An alteration of these parameters in cGAS^-/-^ macrophages after LPS or LPS + DNA was less severe compared with WT cells (Figure 7A–H), indicating the lower impact on energy status and mitochondrial injury of cGAS^-/-^ macrophages. In LPS-stimulated cGAS^-/-^ macrophages, only maximal respiration and *PRKAA2*, not glycolysis activity, mitochondrial abundance (mtDNA, fluorescent staining) and *PRKAA1*, were lower than cGAS^-/-^ control macrophages (Figure 7A–H). These data also supported a reduced impact of LPS on cGAS^-/-^ macrophages compared with WT cells. Additionally, the worsening of maximal respiration in LPS + DNA compared with LPS alone in cGAS^-/-^ macrophages, but not in WT cells (Figure 7A–C), implies a better cell-energy reserve in cGAS^-/-^ macrophages, which may have been responsible for the less severe LPS responses. With DNA activation alone, there was a decrease in maximal respiration (similar to that observed with LPS stimulation), without an increase in glycolysis in both WT and cGAS^-/-^ macrophages (Figure 7A–F). However, the reduced mitochondrial abundance (mtDNA, fluorescent staining) was demonstrated only in WT macrophages (Figure 7A–F), supporting an impact of cGAS activation on mitochondrial injury.

## 3. Discussion

### 3.1. Less Severe Sepsis in cGAS Deficient Mice Due to Reduced Pro-inflammatory Responses

Inflammation-induced cell death (apoptosis and NETosis—neutrophil-extracellular traps induced cell death) [50] and microbial breakdown as a result of sepsis increase levels of serum cfDNA, host DNA and microbial DNA, which could activate the cytosolic cGAS receptor [22,51]. Here, serum cfDNA levels in cGAS^-/-^ and WT mice after CLP and LPS were similar, with higher levels of serum cytokines observed in WT mice, implying cGAS-activated hyperinflammation in WT mice despite a similar insult (cfDNA). The less severe sepsis in cGAS^-/-^ mice compared with WT mice supported previous reports of less severe CLP in mice with cGAS downstream signaling deficiency (STING and IRF-3) [23,45]. Interestingly, serum cfDNA in CLP consists of host DNA and bacterial DNA, while cfDNA in the LPS model consists mainly of host cell cfDNA. Indeed, bacterial DNA (and endotoxemia) in CLP mice is responsible from bacteremia, and gut bacterial translocation causes endotoxemia in CLP mice [52,53], while levels of microbial DNA in the LPS model might be lower because bacteremia is undetectable in the LPS model [47,54]. Then, the less profound CLP severity and the less severe responses against LPS injection in cGAS^-/-^ mice compared with WT imply an impact of cfDNA from host cells and organisms (in the CLP model) and cfDNA mainly from host cells (in the LPS model), respectively. Nevertheless, our data support sepsis hyperinflammation through cGAS activation by either host or microbial DNA.

### 3.2. The Co-Presentation of LPS with Bacterial DNA Enhanced Wildtype Macrophage Responses through M1 Polarization and cGAS Upregulation

On account of (i) a severe clinical presentation of sepsis in the CLP model [55], (ii) a potent inflammatory activation of CpG DNA (bacteria) compared with host DNA [28] and (iii) the coexistence of LPS and cfDNA in the serum of CLP mice, macrophages were stimulated by LPS with and without bacterial DNA. Indeed, synergy between LPS and bacterial DNA with respect to inflammation was demonstrated in WT macrophages, as has been previously reported [28,29], through M1 polarization (*iNOS* and *IL-1β* expression), *cGAS* (with *IFN-γ*) upregulation and cGAMP (a secondary messenger from cGAS activation). In DNA-stimulated WT macrophages, *cGAS*, *IFN-γ* and cGAMP also increased, as was observed with LPS stimulation, though with a less prominent M1 polarization (mild upregulation of only *iNOS*), which resulted in the lower supernatant cytokines. Notably, *cGAS* and *IFN-γ* upregulation after LPS stimulation, through LPS-activated cfDNA, has been demonstrated previously [39]. Due to the non-different *TLR-4* expression between LPS versus LPS + DNA in WT macrophages, the synergy of LPS and DNA was less likely induced through TLR-4, despite the possibility of TLR-4 stimulation by cfDNA [10]. This is perhaps due to TLR-4 activation by DNA having been bypassed with the transfection process. In stimulated cGAS^-/-^ macrophages (LPS, DNA or LPS + DNA), there were less prominent inflammatory markers, including supernatant cytokines (TNF-α and IL-6) and several mediators (*NFκB, IFN-γ, IL-1β* and cGAMP), with higher anti-inflammatory markers (*FIZZ-1* and *Arg-1*) compared with WT cells.

### 3.3. Less Mitochondrial Damage in cGAS Deficient Macrophages after LPS Stimulation a Possible Effect of Mitochondrial DNA in Sepsis

**Figure 8:**
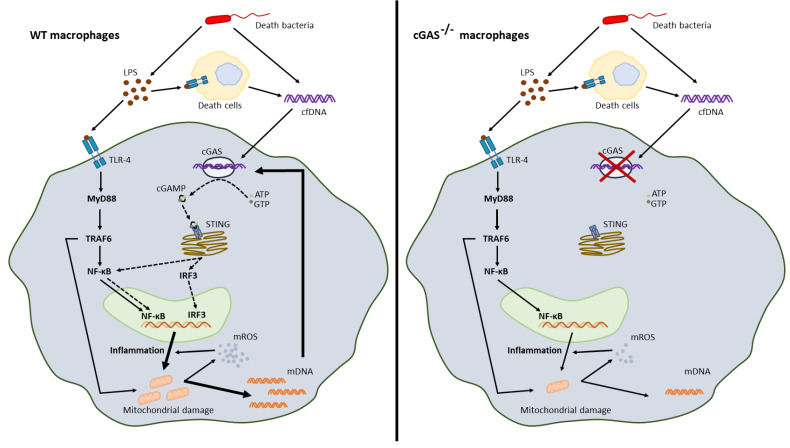
The working hypothesis demonstrates the synergy between lipopolysaccharide (LPS) and cell free DNA (cfDNA); bacterial DNA and host DNA (from LPS-induced cell death), through TLR-4 and cyclic GMP–AMP synthase (cGAS). In WT macrophages, LPS with cfDNA activated a severe inflammation through TLR-4 (MyD88, TRAF6 and NF-κB) and cGAS (cGAMP generation, STIN, and IRF-3), causing mitochondrial DNA (mtDNA) release that also activated cGAS and induced further inflammation (**left-hand image**). Additionally, mitochondrial reactive oxygen species (mtROS) also increased inflammation. Without cGAS, there was a less prominent inflammation that caused less mtDNA to be released and an absence of mtDNA-induced cGAS resulted in lower inflammatory responses in cGAS deficient macrophages compared with WT cells (**right-hand image**). Hence, a vicious cycle of cGAS activation-induced mitochondrial damage ensued, mtDNA further facilitating inflammation-induced mitochondrial injury which we suggest might have enhanced LPS responses. ATP, adenosine triphosphate; GTP, guanosine triphosphate; cGAMP, cyclic guanosine–adenosine monophosphate (GMP–AMP); MyD88, myeloid differentiation primary response 88; TRAF6, TNF receptor associated factor 6; NF-κB, nuclear factor kappa-light-chain-enhancer of activated B cells; IRF3, interferon regulatory factor 3; STING, stimulator of interferon genes; mtROS, mitochondrial reactive oxygen species.

Mitochondria are important for ATP production and mitochondrial free DNA in serum is associated with sepsis severity [40,56,57], partly on account of cGAS activation [58]. Here, impacts of mitochondria on LPS-stimulated WT macrophages were demonstrated by (i) the presence of mitochondria-associated-genes, as determined by RNA sequencing analysis, and (ii) enhanced glycolysis with decreased maximal respiration, as determined with extracellular flux analysis [59,60]. In macrophages, LPS induces inflammatory cytokines, partly, through accelerated glycolysis, as glycolysis is one of the main sources of energy in M1 polarized macrophages [47]. Also, LPS elevates macrophage cytosolic mtDNA that are supported by the downregulated-AMPK, a mitophagy inducer inhibiting mtDNA release [61], and mtDNA-activated cGAS [62], as has previously been reported. Surprisingly, bacterial DNA also blocked mitochondrial functions, as occurred with LPS activation; however, DNA stimulation did not enhance macrophage glycolysis, which was possibly related to a lower cytokine production in DNA stimulation than the LPS responses [47]. In LPS-activated cGAS^-/-^ macrophages, glycolysis elevation and reduction in maximal respiration were less prominent than LPS-activated WT cells without an alteration in mitochondrial abundance. The less prominent mitochondrial damage after LPS stimulation alone (without DNA) in cGAS^-/-^ macrophages compared with WT cells implied an impact of LPS-induced cfDNA from other dead cells in the experiments (Figure 8); however, an influence of self-DNA was not directly tested in vitro in the current study. It may be that LPS and cfDNA (from bacteria and host cells) additively induce profound inflammation causing mitochondrial breakdown and mtDNA exposure to cGAS, which further enhances inflammatory responses in WT macrophages (Figure 8). Without cGAS, the inflammatory responses are less prominent. In vivo, bacterial cfDNAs could be delivered into cytosol and stimulate cytosolic cGAS by several pathways, including high mobility group box-1 and bacterial outer membrane vesicles [25]. Hence, a cGAS inhibitor [63] might be an interesting candidate for anti-inflammatory sepsis treatment. It should be noted that only male mice were used in our experiments given the possibility that gender differences may have an influence on sepsis. Further studies would be valuable here.

## 4. Materials and Methods

### 4.1. Animal

The animal study protocol (025/2563), following the US National Institutes of Health (NIH) animal care and use protocol, were approved by the Institutional Animal Care and Use Committee of the Faculty of Medicine, Chulalongkorn University. Wildtype C57BL/6J (WT) mice were purchased from Nomura Siam (Pathumwan, Bangkok, Thailand) and cGAS knockout (cGAS^-/-^) mice in C57BL/6J background were kindly provided by Paludan (Aarhus University, Aarhus, Denmark). Only male 8 wk-old mice, weighing approximately 20–22 g, were used. The mice were housed in standard clear plastic cages (3–5 mice per cage), had free access to water and food (SmartHeart Rodent; Perfect Companion Pet care, Bangkok, Thailand) and were subject to light/dark cycles of 12/12 h in 22 ± 2 °C, with 50 ± 10% relative humidity and thick paper stripes for environmental enrichment.

### 4.2. Animal Model

Cecal ligation and puncture (CLP) was performed as previously described [64]. Briefly, the mice were anesthetized with an isoflurane (Piramal Critical Care, Bethlehem, PA) and the ceca were ligated and punctured twice with a 21-gauge needle. In sham operated mice, all the surgical procedures were performed except for ligation and puncture of the cecum. The animals were returned to their cages where they had free access to food and water. Mice were sacrificed at 24 h via cardiac puncture under isoflurane anesthesia. The internal organs (including livers, kidneys and lungs) were collected and stored in 10% formalin for histology. Blood was collected at 6 h through tail vein nicking and at 24 h post-CLP by cardiac puncture under isoflurane anesthesia and kept at −80 °C until analyzed. For lipopolysaccharides (LPS) injection, mice were given intraperitoneal (IP) injections of 10 mg/kg of LPS from *Escherichia coli* 026: B6 (Sigma-Aldrich, St. Louis, MO, USA). Blood collection through the tail vein was performed at 3 days before LPS injection (0 h) and at 1 and 3 h post-LPS. Mice were sacrificed at 6 h post LPS injection by cardiac puncture under isoflurane anesthesia.

### 4.3. Blood Sample Analysis

Serum endotoxin (LPS) was measured using the Limulus Amebocyte lysate test (Associates of Cape Cod, East Falmouth, MA, USA) and values of LPS < 0.01 EU/mL were recorded as 0 due to the limitation of the standard curve. Kidney injury (serum creatinine and blood urea nitrogen) and liver damage (alanine transaminase—ALT) were determined by QuantiChrom Creatinine-Assay (DICT-500), Urea-assay (DIUR-500) and EnzyChrom ALT assay (EALT-100) (BioAssay, Hayward, CA, USA). Serum cytokines and cell-free DNA (cfDNA) were measured by enzyme-linked immunosorbent assay (ELISA) (Invitrogen, San Diego, CA, USA) and Quanti PicoGreen assay (Sigma-Aldrich), respectively. For peripheral blood leukocytes, blood was mixed with 3% volume by volume (*v*/*v*) of acetic acid for red blood cell lysis in a ratio of blood and acetic acid at 1:20 by volume before counting with hemocytometer. In addition, Wright-stained blood smear was determined for the percentage of neutrophils and lymphocytes. The total number of these cells was calculated by the total count from hemocytometer multiplied by the percentage of cells from the Wright-stained slide.

### 4.4. Histology

The semi-quantitative evaluation of kidney, liver and lung histology on paraffin-embedded slides was performed after 10% neutral buffered formalin fixation, followed by hematoxylin and eosin (H&E) staining at 200× magnification in 20 randomly selected fields for each animal. Regarding lung injury, semi-quantitative evaluation was made for alveolar hemorrhage, alveolar congestion, neutrophil infiltration and alveolar wall thickness using the following score: 0 points, no injury in the observed field; 1 point, injury up to 25%; 2 points, injury up to 50%; 3 points, injury up to 75%; 4 points, injury to the entire field [65]. The liver histological score was the sum of hepatocyte injury characteristics (cytoplasmic color fading, vacuolization, nuclear condensation, nuclear fragmentation, nuclear fading and erythrocyte stasis) ranging from 0 to 5 multiplied by grades of damage: 0, no injury; 1, mild injury; 2, moderate injury; 3, severe injury. This was in accordance with [66]. Renal injury score was defined by area of the injury (tubular epithelial swelling, loss of brush border, vacuolar degeneration, necrotic tubules, cast formation and desquamation) using the following score: 0, area <5%; 1, area 5–10%; 2, area 10–25%; 3, area 25–50%; 4, area >50% [64,65,67,68]. Spleen apoptosis was detected by immunohistochemistry with anti-activated caspase 3 antibody (Cell Signaling Technology, Beverly, MA, USA), examined in whole section in 200× fields and expressed as positive cells per high-power field [64,67,69].

### 4.5. Macrophage Preparation

Macrophages were derived from bone marrow as previously described [67,70]. Briefly, bone marrow from femurs and tibias was collected by centrifugation at 6000 rpm for 4 °C and incubated for 7 days with Dulbecco’s Modified Eagle Medium (DMEM) supplemented with 10% fetal bovine serum (FBS), 1% penicillin/streptomycin and 4-(2-hydroxyethyl)-1-piperazineethanesulfonic acid (HEPES) with sodium pyruvate in a humidified 5% CO_2_ incubator at 37 °C. Conditioned media of the L929 cell line, containing macrophage-colony stimulating factor, at 20% weight by volume (*w*/*v*), was used to induce macrophages from the pluripotent stem cells.

### 4.6. Cytokines, 2’3’-cGAMP and Gene Expression

For *E. coli* DNA extraction, *E. coli* (ATCC 9637) (American Type Culture Collection, Manassas, VA, USA) in Luria-Bertani (LB) broth were isolated DNA using a Tissue Genomic DNA extraction mini kit (Favorgen Biotech, Wembley, WA, Australia) and quantified by NanoDrop ND-100 (Thermo Scientific). Then, DNA transfection with 5 ug/uL of *E. coli* DNA, using lipofectamine 2000 (2.5 µL in OptiMEM-I 100 µL) (Invitrogen), with macrophages from WT and cGAS^-/-^ mice at 1 × 10^5^ cells/well at 37 °C for 40 min, was performed before an addition of LPS (100 ng/mL) using *Escherichia coli* 026: B6 LPS (Sigma-Aldrich) or media control for the LPS plus DNA (LPS + DNA) group or bacterial DNA transfection alone (DNA), respectively. In LPS alone and the media control, lipofectamine 2000 without DNA was also used for a proper control of the experiments. After 24 h incubation, supernatant cytokines (TNF-α, IL-6 and IL-10) and cyclic guanosine monophosphate–adenosine monophosphate (2’3’-cGAMP) were determined by enzyme-linked immunosorbent assay (ELISA) from Invitrogen and Cayman Chemical (Ann Arbor, MI, USA), respectively. The cells were collected in parallel to evaluate gene expression by real-time quantitative reverse transcription polymerase chain reaction (qRT–PCR), as previously described [47,54]. Accordingly, total RNA was prepared by Trizol, quantified by NanoDrop ND-1000 (Thermo Fisher Scientific), converted into cDNA by Reverse Transcription System) and qPCR was performed using SYBR Green system (Applied Biosystem, Foster City, CA, USA). cDNA template and target primers based on the ΔΔCT method (2^−∆∆Ct^) with the *β-actin* housekeeping gene were used. The primers for cytokines (*TNF-α*, *IL-6* and *IL-10*), M1 macrophage polarization (*iNOS* and *IL-1β*), M2 macrophage polarization (*FIZZ-1*, *Arginase-1* and *TGF-β*), inflammatory signals (*TLR-4*, *cGAS* and *NF-κB*) and cell energy status (*PRKAA1*—protein kinase AMP-activated catalytic subunit alpha 1—and *PRKAA2*—protein kinase AMP-activated catalytic subunit alpha 2) are listed in Table 1.

### 4.7. Mitochondrial Evaluation and Extracellular Flux Analysis

Mitochondrial enumeration was evaluated by mitochondrial DNA (mtDNA) quantification using a Tissue Genomic DNA extraction mini kit (Favorgen Biotech, Wembley, WA, Australia) with NanoDrop ND-100 (Thermo Fisher Scientific) by ΔΔCT method using β2-microglobulin (β2M) normalization as previously described [46]. In parallel, mitochondrial biogenesis was analyzed by MitoTracker, using 200 nM of Mitotracker Red CMxROS (Molecular Probes, Inc., Eugene, OR, USA), that was incubated at 37 °C for 15 min before fixing with cold methanol at −20 °C and measured by microplate reader at excitation OD579 nm and emission OD599 nm, as previously described [46]. Moreover, cell energy status was determined by extracellular flux analysis using Seahorse XFp Analyzers (Agilent, Santa Clara, CA, USA) with oxygen consumption rate (OCR) and extracellular acidification rate (ECAR) representing mitochondrial function (respiration) and glycolysis activity, respectively, as previously described [47]. The stimulated macrophages at 1 × 10^5^ cells/well were incubated by Seahorse media (DMEM complemented with glucose, pyruvate and L-glutamine) (Agilent, 103575-100) for 1 h before activation by different metabolic interference compounds, including oligomycin, carbonyl cyanide-4-(trifluoromethoxy)-phenylhydrazone (FCCP) and rotenone/antimycin A, for OCR evaluation. In parallel, glycolysis stress tests were performed using glucose, oligomycin and 2-Deoxy-d-glucose (2-DG) for ECAR measurement. The data were analyzed by Seahorse Wave 2.6 software based on the following equations: (i) maximal respiration = OCR between FCCP and rotenone/antimycin A − OCR after rotenone/antimycin A; (ii) maximal glycolysis (glycolysis capacity) = ECAR between oligomycin and 2-DG − ECAR after 2-DG.

### 4.8. RNA Sequencing

RNA sequencing analysis was performed to determine the influence of mitochondria on macrophages after LPS stimulation. Briefly, macrophages after the 24 h activation by LPS (Sigma-Aldrich) (100 ng/mL) or control media were collected for RNA extraction using an RNeasy mini kit (Qiagen). The independent triplicated experiments were performed to achieve three samples per group. Then, the samples were processed with the RNA sequencing BGISEQ-50 platform produced by the BGI Company, as previous published [48]. Briefly, sequencing quality was determined using FastQC and the abundance transcripts were quantified by Kallisto before converting into genes with the Tximport r package. The read count data were determined by differential gene expression (DEGs) using edgeR. The fold-change (FC) 1 and -1 were used as the cutoff to choose DEGs. The up- and downregulated genes were analyzed in a biological process (GO Term) using Enrichr. The gene lists that were associated with mitochondrial oxidative phosphorylation and ATP synthesis were selected based on the GO Term of DEGs and the heat maps were generated by pheatmap R package.

### 4.9. Statistical Analysis

All data was analyzed using the Statistical Package for Social Sciences software (SPSS 22.0, SPSS Inc., Chicago, IL, USA) and Graph Pad Prism version 7.0 software (La Jolla, CA, USA). The results were presented as mean ± standard error (SE). The differences between groups were examined for statistical significance by Student’s *t*-test or one-way analysis of variance (ANOVA) with Tukey’s test for the analysis of two groups or multiple groups comparison, respectively. The time-point experiments were analyzed by repeated measures of ANOVA. A *p* value < 0.05 was considered statistically significant.

## 5. Conclusions

In conclusion, the less severe sepsis that was observed in CLP and LPS models in cGAS^-/-^ mice compared with WT mice was possibly due to a synergy between LPS and cfDNA that increased mitochondrial dysfunction and inflammatory responses. Anti-inflammatory sepsis treatment by a cGAS inhibitor might be a promising therapeutic strategy and should be investigated further.

## Figures and Tables

**Table 1:** List of primers used in the study.

Primers	Forward	Reverse
AMPK (PRKAA1)	5’ -AGAGGGCCGCAATAAAAGAT- 3’	5’ -TGTTGTACAGGCAGCTGAGG- 3’
AMPK (PRKAA2)	5’ -TGGCTGCCTTCTTATGCTTT- 3’	5’ -GCTTTGAAACGGCTTCTCAC- 3’
Arginase-1 (Arg-1)	5’ -CTTGGCTTGCTTCGGAACTC- 3’	5’ -GGAGAAGGCGTTTGCTTAGTTC- 3’
cyclic GMP–AMP syntase pair 9 (cGAS pair 9)	5’ -ATGTGAAGATTTCYGCTCCTAATGA- 3’	5’ -GAAATGACTCAGCGGATTTCCT- 3’
Inducible nitric oxide synthase (iNOS)	5’ -ACCCACATCTGGCAGAATGAG- 3’	5’ -AGCCATGACCTTTCGCATTAG- 3’
Interferon gamma (IFN-γ)	5’ -ACTGACTTGAATGTCCAACGCA- 3’	5’ -ATCTGACTCCTTTTTCGCTTCC- 3’
Interleukin-10 (IL-10)	5’ -GCTCTTACTGACTGGCATGAG- 3’	5’ -CGCAGCTCTAGGAGCATGTG- 3’
Interleukin-1β (IL-1β	5’ -GAAATGCCACCTTTTGACAGTG- 3’	5’ -TGGATGCTCTCATCAGGACAG- 3’
Interleukin-6 (IL-6)	5’ -TACCACTTCACAAGTCGGAGGC- 3’	5’ -CTGCAAGTGCATCATCGTTGTTC- 3’
Mitochrondia DNA (mtDNA)	5’ -CGTACACCCTCTAACCTAGAGAAGG- 3’	5’ -GGTTTTAAGTCTTACGCAATTTCC- 3’
Nuclear factor-κB (NF-κB)	5’ -CTTCCTCAGCCATGGTACCTCT- 3’	5’ -CAAGTCTTCATCAGCATCAAACTG- 3’
Resistin-like molecule-α (FIZZ-1)	5’ -GCCAGGTCCTGGAACCTTTC- 3’	5’ -GGAGCAGGGAGATGCAGATGA- 3’
Toll like receptor 4 (TLR-4)	5’ -GGCAGCAGGTGGAATTGTAT- 3’	5’ -AGGCCCCAGAGTTTTGTTCT- 3’
Transforming Growth Factor-β (TGF-β)	5’ -CAGAGCTGCGCTTGCAGAG- 3’	5’ -GTCAGCAGCCGGTTACCAAG- 3’
Tumor necrosis factor α (TNF-α)	5’ -CCTCACACTCAGATCATCTTCTC- 3’	5’ -AGATCCATGCCGTTGGCCAG- 3’
β2-microglobulin (β2M)	5′ -TTCTGGTGCTTGTCTCACTGA- 3′	5′ -CAGTATGTTCGGCTTCCCATTC- 3’
β-actin	5’ -CGGTTCCGATGCCCTGAGGCTCTT- 3’	5’ -CGTCACACTTCATGATGGAATTGA- 3’

## Data Availability

Not applicable.

## References

[1] Singer M., Deutschman C.S., Seymour C.C., Shankar-Hari M., Annane D., Bauer M., Bellomo R., Bernard G.R., Chiche J.-D., Coopersmith C.C. (2016). The Third International Consensus Definitions for Sepsis and Septic Shock (Sepsis-3). JAMA.

[2] Rajaee A., Barnett R., Cheadle W.G. (2018). Pathogen- and Danger-Associated Molecular Patterns and the Cytokine Response in Sepsis. Surg. Infect..

[3] Hotchkiss R.S., Moldawer L.L., Opal S.M., Reinhart K., Turnbull I.R., Vincent J.L. (2016). Sepsis and septic shock. Nat. Rev. Dis. Primers.

[4] Mosser D.M., Hamidzadeh K., Goncalves R. (2020). Macrophages and the maintenance of homeostasis. Cell. Mol. Immunol..

[5] Kotas M.E., Matthay M.A. (2018). Mesenchymal stromal cells and macrophages in sepsis: New insights. Eur. Respir. J..

[6] Da Silva F.P., Nizet V. (2009). Cell death during sepsis: Integration of disintegration in the inflammatory response to overwhelming infection. Apoptosis.

[7] Rhodes A., Wort S.J., Thomas H., Collinson P., Bennett E.D. (2006). Plasma DNA concentration as a predictor of mortality and sepsis in critically ill patients. Crit. Care.

[8] Martins G.A., Kawamura M.T., Carvalho M.D.G.D.C. (2006). Detection of DNA in the Plasma of Septic Patients. Ann. New York Acad. Sci..

[9] Miyake K., Onji M. (2013). Endocytosis-free DNA sensing by cell surface TLR9 in neutrophils: Rapid defense with autoimmune risks. Eur. J. Immunol..

[10] Behnen M., Leschczyk C., Moller S., Batel T., Klinger M., Solbach W., Laskay T. (2014). Immobilized immune complexes induce neutrophil extracellular trap release by human neutrophil granulocytes via FcgammaRIIIB and Mac-1. J. Immunol..

[11] Margolis S.R., Wilson S.C., Vance R.E. (2017). Evolutionary Origins of cGAS-STING Signaling. Trends Immunol..

[12] Zhao Q., Wei Y., Pandol S.J., Li L., Habtezion A. (2018). STING Signaling Promotes Inflammation in Experimental Acute Pancreatitis. Gastroenterology.

[13] Torralba D., Baixauli F., Villarroya Beltri C., Fernández Delgado I., Latorre Pellicer A. (2018). Priming of dendritic cells by DNA containing extracellular vesicles from activated T cells through antigen driven con tacts. Nat. Commun..

[14] Ablasser A., Gulen M.F. (2016). The role of cGAS in innate immunity and beyond. J. Mol. Med..

[15] Zhou C.M., Wang B., Wu Q., Lin P., Qin S.G., Pu Q.Q., Yu X.-J., Wu M. (2021). Identification of cGAS as an innate immune sensor of extracellular bacterium Pseudomonas aeruginosa. Iscience.

[16] Huang L.S., Hong Z., Wu W., Xiong S., Gao X., Rehman J., Malik A.B. (2020). mtDNA Activates cGAS Signaling and Suppresses the YAP-Mediated Endothelial Cell Proliferation Program to Promote Inflammatory Injury. Immunity.

[17] Cheng Z.L., Dai T., He X.L., Zhang Z.K., Xie F., Wang S., Zhang L., Zhou F. (2020). The interactions between cGAS-STING pathway and pathogens. Signal Transduct. Target. Ther..

[18] Ruiz-Moreno J.S., Hamann L., Shah J.A., Verbon A., Mockenhaupt F.P., Puzianowska-Kuznicka M., Naujoks J., Sander L.E., Witzenrath M., Cambier J.C. (2018). The common HAQ STING variant impairs cGAS-dependent antibacterial responses and is associated with susceptibility to Legionnaires’ disease in humans. PLoS Pathog..

[19] Ahn J., Barber G.N. (2019). STING signaling and host defense against microbial infection. Exp. Mol. Med..

[20] Watson R.O., Bell S., MacDuff D.A., Kimmey J.M., Diner E.J., Olivas J., Vance R.E., Stallings C.L., Virgin H., Cox J.S. (2015). The Cytosolic Sensor cGAS Detects Mycobacterium tuberculosis DNA to Induce Type I Interferons and Activate Autophagy. Cell Host Microbe.

[21] Xia P., Wang S., Gao P., Gao G., Fan Z. (2016). DNA sensor cGAS-mediated immune recognition. Protein Cell.

[22] Decout A., Katz J.D., Venkatraman S., Ablasser A. (2021). The cGAS–STING pathway as a therapeutic target in inflammatory diseases. Nat. Rev. Immunol..

[23] Heipertz E.L., Harper J., Walker W.E. (2017). STING and TRIF Contribute to Mouse Sepsis, Depending on Severity of the Disease Model. Shock.

[24] Hu Q., Ren H., Li G., Wang D., Zhou Q., Wu J., Zheng J., Huang J., Slade D.A., Wu X. (2019). STING-mediated intestinal barrier dysfunction contributes to lethal sepsis. EBioMedicine.

[25] Cheng Z., Abrams S.T., Austin J., Toh J., Wang S.S., Wang Z., Yu Q., Yu W., Toh C.H., Wang G. (2020). The Central Role and Possible Mechanisms of Bacterial DNAs in Sepsis Development. Mediat. Inflamm..

[26] Gosiewski T., Ludwig-Galezowska A.H., Huminska-Lisowska K., Sroka-Oleksiak A., Radkowski P., Salamon D., Wojciechowicz J., Kus-Slowinska M., Bulanda M., Wolkow P.P. (2016). Comprehensive detection and identification of bacterial DNA in the blood of patients with sepsis and healthy volunteers using next-generation sequencing method—The observation of DNAemia. Eur. J. Clin. Microbiol. Infect. Dis..

[27] Hamaguchi S., Akeda Y., Yamamoto N., Seki M., Yamamoto K., Oishi K., Tomono K. (2015). Origin of Circulating Free DNA in Sepsis: Analysis of the CLP Mouse Model. Mediat. Inflamm..

[28] Cornélie S., Wiel E., Lund N., Lebuffe G., Vendeville C., Riveau G., Vallet B., Ban E. (2002). Cytosine-phosphate-guanine (CpG) motifs are sensitizing agents for lipopolysaccharide in toxic shock model. Intensiv. Care Med..

[29] Yi A.-K., Yoon J.-G., Hong S.-C., Redford T.W., Krieg A.M. (2001). Lipopolysaccharide and CpG DNA synergize for tumor necrosis factor-α production through activation of NF-κB. Int. Immunol..

[30] Sparwasser T., Miethke T., Lipford G., Borschert K., Häcker H., Heeg K., Wagner H. (1997). Bacterial DNA causes septic shock. Nature.

[31] Liao W., Zuo X., Lin G., Zhou Y., Fu Y., Cai S., Wei P.-P., Liu Y.-X., Liu Y., Ma G. (2020). Microbial cell-free DNA in plasma of patients with sepsis: A potential diagnostic methodology. Discov. Med..

[32] Chen P., Li S., Li W., Ren J., Sun F., Liu R., Zhou X.J. (2020). Rapid diagnosis and comprehensive bacteria profiling of sepsis based on cell-free DNA. J. Transl. Med..

[33] Sirivongrangson P., Kulvichit W., Payungporn S., Pisitkun T., Chindamporn A., Peerapornratana S., Pisitkun P., Chitcharoen S., Sawaswong V., Worasilchai N. (2020). Endotoxemia and circulating bacteriome in severe COVID-19 patients. Intensive Care Med. Exp..

[34] Prins J.M., van Deventer S.J., Kuijper E.J., Speelman P. (1994). Clinical relevance of antibiotic-induced endotoxin release. Antimicrob. Agents Chemother..

[35] Rimmele T., Kellum J.A. (2011). Clinical review: Blood purification for sepsis. Crit. Care.

[36] Lilleri D., Gerna G., Furione M., Bernardo M.E., Giorgiani G., Telli S., Baldanti F., Locatelli F. (2007). Use of a DNAemia cut-off for monitoring human cytomegalovirus infection reduces the number of preemptively treated children and young adults receiving hematopoietic stem-cell transplantation compared with qualitative pp65 antigenemia. Blood.

[37] Wu J., Sun L., Chen X., Du F., Shi H., Chen C., Chen Z.J. (2012). Cyclic GMP-AMP Is an Endogenous Second Messenger in Innate Immune Signaling by Cytosolic DNA. Science.

[38] Van der Slikke E.C., Star B.S., van Meurs M., Henning R.H., Moser J., Bouma H.R. (2021). Sepsis is associated with mitochondrial DNA damage and a reduced mitochondrial mass in the kidney of patients with sepsis-AKI. Crit. Care.

[39] Li T., Chen Z.J. (2018). The cGAS-cGAMP-STING pathway connects DNA damage to inflammation, senescence, and cancer. J. Exp. Med..

[40] Timmermans K., Kox M., Scheffer G.J., Pickkers P. (2016). Plasma Nuclear and Mitochondrial DNA Levels, and Markers of Inflammation, Shock, and Organ Damage in Patients with Septic Shock. Shock.

[41] Di Caro V., Walko T.D., Bola R.A., Hong J.D., Pang D., Hsue V., Au A.K., Halstead E.S., Carcillo J.A., Clark R.S.B. (2016). Plasma Mitochondrial DNA—A Novel DAMP in Pediatric Sepsis. Shock.

[42] Farkas J.D. (2020). The complete blood count to diagnose septic shock. J. Thorac. Dis..

[43] Gao J.J., Xue Q., Papasian C.J., Morrison D.C. (2001). Bacterial DNA and Lipopolysaccharide Induce Synergistic Production of TNF-α Through a Post-Transcriptional Mechanism. J. Immunol..

[44] Tan Y., Zanoni I., Cullen T.W., Goodman A.L., Kagan J.C. (2015). Mechanisms of Toll-like Receptor 4 Endocytosis Reveal a Common Immune-Evasion Strategy Used by Pathogenic and Commensal Bacteria. Immunity.

[45] Jiang G.-L., Yang X.-L., Zhou H.-J., Long J., Liu B., Zhang L.-M., Lu D. (2021). cGAS knockdown promotes microglial M2 polarization to alleviate neuroinflammation by inhibiting cGAS-STING signaling pathway in cerebral ischemic stroke. Brain Res. Bull..

[46] Jaroonwitchawan T., Visitchanakun P., Dang P.C., Ritprajak P., Palaga T., Leelahavanichkul A. (2020). Dysregulation of lipid metabolism in macrophages is responsible for severe endotoxin tolerance in FcgRIIB-deficient lupus mice. Front. Immunol..

[47] Dang C.P., Issara-Amphorn J., Charoensappakit A., Udompornpitak K., Bhunyakarnjanarat T., Saisorn W., Sae-Khow K., Leelahavanichkul A. (2021). BAM15, a Mitochondrial Uncoupling Agent, Attenuates Inflammation in the LPS Injection Mouse Model: An Adjunctive Anti-Inflammation on Macrophages and Hepatocytes. J. Innate Immun..

[48] Issara-Amphorn J., Dang C., Saisorn W., Limbutara K., Leelahavanichkul A. (2021). Candida Administration in Bilateral Nephrectomy Mice Elevates Serum (1⟶3)-β-D-glucan That Enhances Systemic Inflammation through Energy Augmentation in Macrophages. Int. J. Mol. Sci..

[49] Wu S., Zou M.H. (2020). AMPK, Mitochondrial Function, and Cardiovascular Disease. Int. J. Mol. Sci..

[50] Bantel H., Schulze-Osthoff K. (2009). Cell death in sepsis: A matter of how, when, and where. Crit. Care.

[51] Hopfner K.P., Hornung V. (2020). Molecular mechanisms and cellular functions of cGAS-STING signalling. Nat. Rev. Mol. Cell Biol..

[52] Leelahavanichkul A., Worasilchai N., Wannalerdsakun S., Jutivorakool K., Somparn P., Issara-Amphorn J. (2016). Gastrointestinal Leakage Detected by Serum (1-->3)-beta-D-Glucan in Mouse Models and a Pilot Study in Patients with Sepsis. Shock.

[53] Amornphimoltham P., Yuen P.S.T., Star R.A., Leelahavanichkul A. (2019). Gut Leakage of Fungal-Derived Inflammatory Mediators: Part of a Gut-Liver-Kidney Axis in Bacterial Sepsis. Dig. Dis. Sci..

[54] Udompornpitak K., Bhunyakarnjanarat T., Charoensappakit A., Dang C.P., Saisorn W., Leelahavanichkul A. (2021). Lipopolysaccharide-Enhanced Responses against Aryl Hydrocarbon Receptor in FcgRIIb-Deficient Macrophages, a Profound Impact of an Environmental Toxin on a Lupus-Like Mouse Model. Int. J. Mol. Sci..

[55] Doi K., Leelahavanichkul A., Yuen P., Star R.A. (2009). Animal models of sepsis and sepsis-induced kidney injury. J. Clin. Investig..

[56] Zhang R.-X., Kang R., Tang D.-L. (2021). STING1 in sepsis: Mechanisms, functions, and implications. Chin. J. Traumatol..

[57] Busani S., De Biasi S., Nasi M., Paolini A., Venturelli S., Tosi M., Girardis M., Cossarizza A. (2020). Increased Plasma Levels of Mitochondrial DNA and Normal Inflammasome Gene Expression in Monocytes Characterize Patients With Septic Shock Due to Multidrug Resistant Bacteria. Front. Immunol..

[58] Barber G.N. (2015). STING: Infection, inflammation and cancer. Nat. Rev. Immunol..

[59] Pålsson-McDermott E.M., O’Neill L.A.J. (2020). Targeting immunometabolism as an anti-inflammatory strategy. Cell Res..

[60] Soto-Heredero G., Heras M.M.G.D.L., Gabandé-Rodríguez E., Oller J., Mittelbrunn M. (2020). Glycolysis—A key player in the inflammatory response. FEBS J..

[61] Bai J., Liu F. (2019). The cGAS-cGAMP-STING pathway: A molecular link between immunity and metabolism. Diabetes.

[62] Fan K., Lin L., Ai Q., Wan J., Dai J., Liu G., Tang L., Yang Y., Ge P., Jiang R. (2018). Lipopolysaccharide-Induced Dephosphorylation of AMPK-Activated Protein Kinase Potentiates Inflammatory Injury via Repression of ULK1-Dependent Autophagy. Front. Immunol..

[63] Chu L., Li C., Li Y., Yu Q., Yu H., Li C. (2021). Perillaldehyde Inhibition of cGAS Reduces dsDNA-Induced Interferon Response. Front. Immunol..

[64] Visitchanakun P., Tangtanatakul P., Trithiphen O., Soonthornchai W., Wongphoom J., Tachaboon S., Srisawat N., Leelahavanichkul A. (2019). Plasma miR-370-3P as a Biomarker of Sepsis-Associated Encephalopathy, the Transcriptomic Profiling Analysis of Microrna-Arrays From Mouse Brains. Shock.

[65] Dang C.P., Leelahavanichkul A. (2020). Over-expression of miR-223 induces M2 macrophage through glycolysis alteration and attenuates LPS-induced sepsis mouse model, the cell-based therapy in sepsis. PLoS ONE.

[66] Yang J., Wu R., Qiang X., Zhou M., Dong W., Ji Y., Marini C.P., Ravikumar T.S., Wang P. (2009). Human Adrenomedullin and Its Binding Protein Attenuate Organ Injury and Reduce Mortality after Hepatic Ischemia-Reperfusion. Ann. Surg..

[67] Visitchanakun P., Saisorn W., Wongphoom J., Chatthanathon P., Somboonna N., Svasti S., Fucharoen S., Leelahavanichkul A. (2020). Gut leakage enhances sepsis susceptibility in iron-overloaded β-thalassemia mice through macrophage hyperinflammatory responses. Am. J. Physiol. Liver Physiol..

[68] Taratummarat S., Sangphech N., Vu C.T.B., Palaga T., Ondee T., Surawut S., Sereemaspun A., Ritprajak P., Leelahavanichkul A. (2018). Gold nanoparticles attenuates bacterial sepsis in cecal ligation and puncture mouse model through the induction of M2 macrophage polarization. BMC Microbiol..

[69] Visitchanakun P., Panpetch W., Saisorn W., Chatthanathon P., Wannigama D.L., Thim-Uam A. (2021). Increased susceptibility to dextran sulfate-induced mucositis of iron-overload β-thalassemia mice, another endogenous cause of septicemia in thalassemia. Clin. Sci..

[70] Bhunyakarnjanarat T., Udompornpitak K., Saisorn W., Chantraprapawat B., Visitchanakun P., Dang C., Issara-Amphorn J., Leelahavanichkul A. (2021). Prominent Indomethacin-Induced Enteropathy in Fcgriib *Defi-cient lupus* Mice: An Impact of Macrophage Responses and Immune Deposition in Gut. Int. J. Mol. Sci..

